# Physicians’ knowledge and practice on death certification in the North West Bank, Palestine: across sectional study

**DOI:** 10.1186/s12913-017-2814-y

**Published:** 2018-01-08

**Authors:** Jamal A. S. Qaddumi, Zaher Nazzal, Allam Yacoub, Mahmoud Mansour

**Affiliations:** 1 Faculty of Medicine and Health Sciences, An-Najah National University, PO box 7, Nablus, Palestine; 2 Department of anesthesia, An-Najah National University Hospital, Nablus, Palestine; 3 Department of general surgery, Palestine medical complex, Ramallah, Palestine

**Keywords:** Physicians’ knowledge, Practice, Death certification

## Abstract

**Background:**

Mortality data are essential for many aspects of everyday public health practices at both national and international levels. Despite the current developments in various aspects of the medical field, the apparent inability of physicians to complete death notification forms (DNF) accurately is still worldwide concern. The aim of this study is to assess the physicians’ knowledge and practice on completing the DNF.

**Methods:**

A self-administered questionnaire was distributed to 200 physicians in governmental and non-governmental hospitals in the North West-Bank in Palestine. Furthermore, a case scenario was included in the questionnaire and physicians were asked to fill the cause of death section. The percentage of errors committed while completing the cause of death section were computed. A Chi square test was used to assess the association between physicians’ characteristics and their responses.

**Results:**

Only 40.6% of the participants completed the cause of death section correctly. The immediate and underlying causes of death were correctly identified by 48.7% and 71.3% of physicians, respectively. Almost one-fifth (17.3%) of physicians wrote the mechanism of death without reporting the underlying cause of death and 14.7% of them reported the sequence of events leading to death incorrectly.

**Conclusions:**

Physicians’ knowledge and practice on completing the DNF is poor and insufficient, which may seriously affect the accuracy of mortality data. Complicated cases, problems in the current design of the DNFs and lack of training were the most common factors contributing to inaccuracy in death certification. We recommend offering periodical training workshops on completing the DNF to all physicians, and developing a manual on completing the DNFs with clear instructions and guidelines.

**Electronic supplementary material:**

The online version of this article (10.1186/s12913-017-2814-y) contains supplementary material, which is available to authorized users.

## Background

Physicians are usually required to report the cause-of-death (COD) sequence on a death notification form [1–3]. Accurate COD reporting is a prerequisite for good quality mortality statistics required for health policy decisions [2]. Physicians have to be familiar with international recommendations for completing the DNF. It is important that the DNF should be completed by the physician who is most fully informed about the last illness of the deceased [3].

Briefly, the national DNF which was structured based on international guidelines [4] is composed of two sections: The demographic characteristics and the COD sections. The COD section consists of two parts: Part I for reporting the chain of events leading directly to death, with the immediate COD on the first line and the underlying COD on the lowest used line, and Part II for reporting all other significant diseases, conditions, or injuries that contributed to death.

Despite of the World Health Organization’s (WHO) efforts on adopting guidelines and updating the format, unfortunately, errors in death notification are common [5], and have been noted worldwide [6]. Many studies assessed the physicians’ knowledge and practice on completing the DNF and showed different types of errors and concluded that the physicians lack adequate knowledge, training and experience for completing death certificates [7]. A study conducted in the USA aimed to examine the experiences and opinions of physician residents in New York City on the accuracy of the COD reporting system; it concluded that most resident physicians believed that the current COD reporting system is inaccurate [8].

Different types of errors could occur at various stages of the COD reporting process, ranging from incomplete notification and using abbreviations, to inaccurate cause and manner of death [9]. One classification method divides the errors into major and minor [10]. Major errors include: (I) Incorrect sequencing where the chain of events leading directly to death are written in an order that is not scientifically appropriate; (II) competing causes of death occur when two or more causally unrelated, etiologically specific diseases are listed in part I of the DNF; (III) no acceptable cause of death is documented when signs, symptoms or ill-defined terms such as old age or severe headache are listed in part I of the DNF; (IV) no underlying cause of death after mechanism; this occurs if no underlying cause of death is reported and the notifying physician only uses mechanism of death or reported mechanism of death with incorrect underlying cause of death or there is no link between them. Minor errors include absence of time intervals for each diagnosis, using abbreviations, writing irrelevant information and illegible handwriting.

The aim of this study is to assess physicians’ knowledge and practice on completing the DNF in order to improve the quality of death certification in Palestine.

## Methods

### Study design and population

A cross-sectional study was conducted to assess the physicians’ knowledge and practice on completing the DNF. The study was conducted during the period Nov to Dec 2013 and targeted all physicians working at the governmental and nongovernmental hospitals in the North West Bank. Physicians not involved in care of in-patients and those working in the departments of radiology, pathology and dermatology, were excluded from the study.

Among the eligible physicians (600)working in 50 governmental and non-governmental hospitals, a sample size of 150 physicians was computed using a confidence level of 95%, a precision of 5%, and a 15% estimated proportion of optimal performance, through simple random sampling. To compensate for the non-response, we added an extra 50 physicians (25% of estimated sample) to the calculated sample size to include a total of 200 physicians.

### Data collection

A self-administered questionnaire, to be completed in the English language, was used to collect data. It was adopted from previous studies [11, 12] and was pre-tested with a convenience sample of 20 physicians from the study population to ensure clarity, time and ease of administration. It included questions related to physicians’ demographic characteristics and qualifications (the physicians’ experience, training and practice in completing death certificates) and physicians’ knowledge of death certificate completion, including the completion of a death certificate case scenario. A case scenario (Additional file 1) was included in the questionnaire and the physicians were asked to complete the cause of death section and then their answers were evaluated in terms of errors in completing the DNF.

Incorrect answers were classified as major and minor errors as described above in the introduction. The data abstraction sheet was constructed by the researchers based on national standards and international guidelines [4] to collect information regarding the errors in completing the COD section in the DNF. Researchers reviewed the DNF independently and any disagreements were resolved by consensus based on discussion.

Statistical Package for the Social Sciences (SPSS) version 20.0 was used for data entry and analysis. Proportions of completeness of COD and errors in reporting the COD section were calculated. A chi square test was used to assess the association between participants’ characteristics and their responses. The level of significance was set at 0.05.

Approval from the An-Najah National University institutional review board and the Palestinian Ministry of Health was obtained and consent forms were signed by physicians before completing the questionnaire. Participants’ privacy and confidentiality of collected data were assured.

## Results

A total of 150 physicians completed and returned the questionnaire giving an overall response rate of 75%. The median age of the participants was 29 years, with ages ranging from 25 to 65 years. Male gender constituted 79.3% of the participants, and a majority of them (63.3%) were working in governmental hospitals. Resident physicians constituted the majority of the sample (63.3%).

The majority of participants (82.7%) reported they had experience in completing the DNF, and only 21.3% of all participants reported that they received previous training for completing the DNF. Regarding their specialty, the sample was distributed almost equally, and about one fourth (26.7%) were graduated from universities in Palestine (Table 1).

**Table 1:** Characteristics of study participants (*N* = 150)

Characteristic	Frequency Number	Percentage (%)
Age	Median 29	Range 25–65
Gender		
Male	119	79.3
Female	31	20.7
Current possession		
Resident	95	63.3
Specialist	55	36.7
Work place		
Governmental	95	63.3
Non-governmental	55	36.7
Graduation country		
Palestine	40	26.7
Other countries	110	73.3
Years since Graduation		
1 year	27	18.0
2–5 years	58	38.9
6–10	30	20.1
11 and more	35	23.0
Specialty		
Medicine	28	18.6
Surgery	54	36.0
Pediatrics	22	14.7
Others	46	30.7
Completed DNF Before		
Yes	124	82.7
No	26	17.3
Trained to complete DNF		
Yes	32	21.3
No	118	78.7

**Fig. 1:**
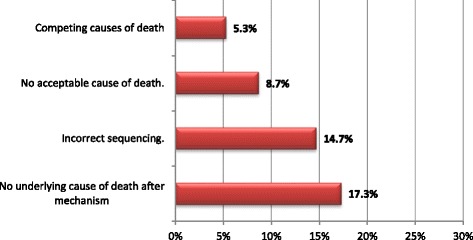
Distribution of Major Errors in the Case Scenario (*n* = 150)

The majority of physicians who had experience in completing the DNF (62%) reported facing difficulties in completing it (Fig. 1). Dealing with complicated cases and the design of the DNF were the most common difficulties reported by physicians (Table 2).

**Table 2:** Distribution of difficulties when completing previous DNF (*N* = 77)

Difficulty type	Frequency Number	Percentage (%)
Dealing with complicated cases	50	64.9
Problem in design of DNF	37	48.1
Lack of training	30	39.0
Did not understand terms	22	28.6
Lack of time	16	20.8

Evaluating the results on the case scenario indicated that only 40.6% of the participants filled the COD section completely correctly. At least one major error was made by 44.3% of the physicians. The most common major error was “No underlying cause of death after mechanism of death” (17.3%), followed by “Incorrect sequence” error (14.7%) (Fig. 1).

**Fig. 2:**
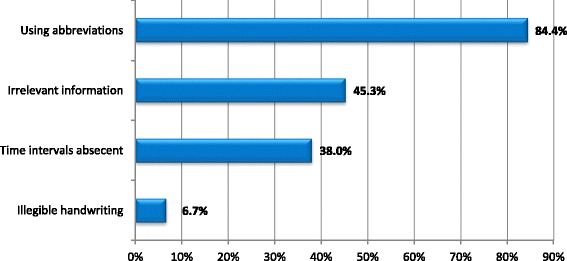
Distribution of Minor Errors in Case Scenario (*n* = 150)

For minor errors, only 15 (10%) of DNF were free of any minor error. The most frequently occurring minor error by physicians was “Using abbreviations and symbols” error (84.7%), followed by “Irrelevant information” error (45.3%) (Fig. 2).

The frequency of major errors in the case scenario part was assessed in relation to physician characteristics. It was evident that female physicians made less major errors than male physicians. Major errors were less often made by specialists compared to resident physicians. Physicians who worked in governmental hospitals made more errors than those who worked in non- governmental hospitals; a “No acceptable cause of death” error was made by 11.6% of governmental hospital physicians compared with 3.6% in non- governmental (*P* = 0.020).

Physicians graduated from countries other than Palestine made more major errors than those graduated from local medical schools. Physicians with experience in completing the DNF made fewer errors than those who never filled a DNF before. (Table 3).

**Table 3:** Association between major errors in case scenario related to Physicians’ characteristics

	Type of error					
	Mechanism of Death		Incorrect sequencing		No acceptable cause of death	
Characteristic	*n*(%)	*P*-Value*	*n*(%)	*P*-Value*	*n*(%)	*P*-Value*
Gender of physician						
Male	22(18.4)	0.464	20(16.8)	0.147	11(9.2)	0.623
Female	4(13.0)		2(6.5)		2(6.5)	
Current possession						
Resident	20(21.0)	0.114	15(17.8)	0.609	9(9.5)	0.644
Specialist	6(11.0)		7(12.7)		4(7.2)	
Work place						
Governmental	17(18.5)	0.727	15(15.8)	0.523	11(11.6)	0.020
Non-governmental	9(16.4)		7(12.7)		2(3.6)	
Graduation Country						
Palestine	4(10.0)	0.306	1(2.5)	0.011	0(0.0)	0.023
Other country	22(20.0)		21(19.2)		13(11.8)	
Graduation year						
≤ 5 years	18(45.0)	0.306	12(32.5)	0.405	7(14.0)	0.775
> 5 years	7(22.1)		7(27.6)		6(18.8)	
Specialty						
Medicine	5(18.0)	0.872	3(10.7)	0.296	1(3.6)	0.101
Surgery	11(20.4)		6(11.1)		2(3.7)	
Pediatric	3(13.6)		6(27.3)		4(18.2)	
Others	7(15.2)		7(20.0)		6(13.0)	
Completed DNF Before						
Yes	17(13.7)	0.035	15(12.0)	0.143	11(8.8)	0.933
No	9(34.6)		7(7.0)		2(7.6)	
Trained to Fill DNF						
Yes	7(22.6)	0.386	3(9.7)	0.378	4(12.9)	0.347
No	19(16.0)		19(16.0)		9(7.6)	

*Chi-square test

## Discussion

Mortality statistics are one of the vital sources of information about health status and many research projects in many countries consider it as one of the most reliable source of health data. The errors in DNF have many significant effects on long term planning of the health systems in the national and international levels [3, 4].

The majority of the present study participants were males (79.3%), which is consistent with the distribution of the physician work force in Palestinian hospitals. More than half of the participants (56.9%) were graduated 5 years ago or less, and this is in agreement with the results that showed that 63.3% of the participants were residents. This may be attributed to the new residency programs being applied in Palestinian hospitals.

Regarding the formal training on completing the DNF, only 21.3% of study participants reported that they had received training; this is consistent with other studies in the region like Qatar, Bahrain and Nigeria where the proportion of trained physicians were 22.7%, 19% and 29% respectively [11–13]. This low percentage of training could show the decreased importance of this subject perceived by Ministry of Health and other related organizations.

Surprisingly, only 40.7% of participants reported that they need to be trained on how to complete the DNF. The physician need for training in completing the DNF was addressed by many studies where variable results were obtained; 27.4% of physicians in Qatar reported a need for further training compared to 77.6% physicians in Bahrain [11, 12]. Explanations could be that the participants didn’t receive regular feedback on their DNF performance, so they might have believed that they already had the enough experience and practice on completing the DNF, and they might not be sufficiently aware of their errors and/or the importance of completing the DNF accurately.

Among the participants who had past experience on completing the DNF, about 62% of them reported previous difficulties during completing it. The “complicated cases” were the most common difficulty that the participants faced when completing a DNF (64.9%); this is similar to what has been reported in a study conducted in Qatar [12]. This could be attributed to the poor documentation system in most of the hospitals where electronic filing is not being introduced yet; this makes physicians unable to extract complete medical information about the case. This can also explain why governmental hospital physicians did more errors in completion the DNF as they faced more complicated cases and have workloads with shortages of staff. “Problem in design of DNF” was the second most common difficulty reported by the participants. It is noticed that the clinical section of the national DNF deviates substantially from the WHO recommendations. It is not clearly distinguished from the whole document, especially the poor distinction between Part 1 and Part 2, which is a major problem for both certifiers and those producing statistics.

For individual errors observed in the case scenario, this study found that immediate cause of death was identified incorrectly by 51.3% of participants, which is lower than reported by other similar studies [12, 14]. Terms that describe the mechanism of death (mostly cardio-respiratory arrest) were filled incorrectly instead of the “Immediate cause of death” in almost half of the case scenarios (49.3%). Underlying cause of death was identified correctly by 71.3% of the participants compared to 58% of Qatari physicians [12].

We expect the results obtained in this study to have underestimated the errors committed by physician in their real practice. In another study [15] conducted by the same authors, 547 real DNF were randomly selected from the primary health care directorates in the North West Bank to assess their quality and evaluate the most common type of error. In general, the errors observed in the case scenario in this study are much less than those observed in the real DNFs. This divergence may have different explanations: first, the physicians may have given more attention to answering the questionnaire (approaching it as an exam) than they do when completing an actual DNF. Second, being a self administrative questionnaire may have allowed physicians a chance to review the topic to provide “ideal” answers, or ask help from expert physicians in answering the questions. Finally, the case scenario that was included in the questionnaire could not have resembled the complexity of real life situations where different factors play a role in the physicians’ ability to accurately identify different causes, including the patient’s medical history, the quality of the medical records, and the length of time that the physician has been following the patients’ condition prior to death. In addition to that, the circumstances in which the case scenario was filled give more time for the physician to think about the case compared with circumstances when he filled the actual DNF.

## Conclusion

The study findings imply the need to increase physicians’ awareness about the importance of DNF and their implications on improving their practice. This could be through offering training workshops on DNF completion and developing a manual on completing the DNF with clear instructions and guidelines. Medical schools should emphasize this issue and highlight its important in their curriculum. Improve the process of documentation in the health care institutes to increase the amount of available information about the patient in the hands of physicians. Finally, revision of the national DNF is needed to simplify it and make it consistent with the WHO recommendation. Thus, completing the DNF properly will improve accuracy of the national mortality report and, consequently, the evidence-based decision making.

## Additional files

Additional file 1: Case scenario and its model answer. (DOCX 196 kb)

## Abbreviations

COD: Cause of death
DNF: Death notification form
IRB: Institutional review board
MOH: Ministry Of Health
N(n): Number
SPSS: Statistical package for the social sciences
WHO: World Health Organization
