# Supplementary material for: Physicians’ knowledge and practice on death certification in the North West Bank, Palestine: across sectional study
Source: BMC Health Serv Res. 2018 Jan 8;18:8. doi: 10.1186/s12913-017-2814-y (PMC5759221; doi:10.1186/s12913-017-2814-y)

Additional file 1 Case scenario and its model answer

| A 35 year old male was admitted with severe shortness of breath of 10 hours duration. He has a history of right leg pain and swelling for 10 days previously. He visited a doctor who diagnosed him with deep vein thrombosis (DVT). He has takeen drugs for asthma since 2004 and he was diagnosed as protein C deficiency 10 years ago. One hour after admission he was arrested and died after 30 minutes of resuscitation trial |
| --- |


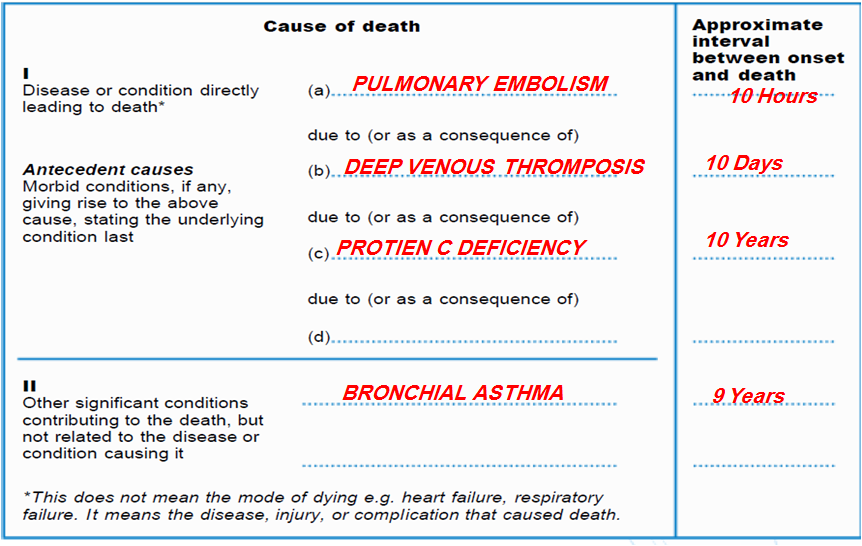

Supplement: Supplementary file 1 — Case scenario and its model answer. (DOCX 196 kb) [file 12913_2017_2814_MOESM1_ESM.docx]
